# A highly accurate metadynamics-based Dissociation Free Energy method to calculate protein–protein and protein–ligand binding potencies

**DOI:** 10.1038/s41598-022-05875-8

**Published:** 2022-02-07

**Authors:** Jing Wang, Alexey Ishchenko, Wei Zhang, Asghar Razavi, David Langley

**Affiliations:** Arvinas, Inc., 5 Science Park, New Haven, CT 06511 USA

**Keywords:** Biochemistry, Biophysics, Biotechnology, Chemical biology, Computational biology and bioinformatics, Drug discovery, Structural biology, Medical research, Molecular medicine, Chemistry, Mathematics and computing, Physics

## Abstract

Although seeking to develop a general and accurate binding free energy calculation method for protein–protein and protein–ligand interactions has been a continuous effort for decades, only limited successes have been obtained so far. Here, we report the development of a metadynamics-based procedure that calculates Dissociation Free Energy (DFE) and its application to 19 non-congeneric protein–protein complexes and hundreds of protein–ligand complexes covering eight targets. We achieved very high correlations in comparison to experimental binding free energies for these diverse sets of systems, demonstrating the generality and accuracy of the method. Since structures of most proteins are available owing to the recent success of prediction by artificial intelligence, a general free energy method such as DFE, combined with other methods, can make structure-based drug design a widely viable and reliable solution to develop both traditional small molecule drugs and biologic drugs as well as PROTACS.

## Introduction

A general method able to accurately calculate binding affinities of protein–protein complexes (PPC) and protein–ligand complexes (PLC) would be highly empowering for rational design of biologic and small molecule drugs. (“Ligand” refers to small molecule). It would also offer researchers a theoretical tool to study processes involving molecular recognition and interaction. Although development of such a method has been the focus of continuous effort for decades, only limited successes have been achieved. For example, empirical functions were developed to be used in docking codes, which are critical to making the docking codes work but usually poor in predicting binding free energies. Molecular Mechanics Poisson-Boltzmann/Surface Area methods and other similar methods were sometimes successful in generating binding free energies for specific systems^1–3^. The potential of mean force approaches which rely on physical pathways to move a binding partner relative to another in a complex between bound and unbound states in molecular dynamics simulations were fruitful in analyzing absolute binding free energies of several biological complexes^4–6^. Funnel-Metadynamics might be one of the most elegant methods that has been developed so far^7–9^, but it was designed only for PLC and not for PPC. Another elegant approach is Free Energy Perturbation (FEP) which generates rigorous relative free energies^10–12^, but its applicability was limited to calculation of relative binding free energy changes among congeneric series of ligands, and the relative free energy changes resulting from point mutations of proteins^13–15^. In this manuscript, we report the development of a metadynamics-based procedure which brings us much closer to being able to predict protein–protein and protein–ligand binding free energies as well as other types of binding free energies, with demonstrated generality and high accuracy.

The procedure to be reported in this paper generates Dissociation Free Energies (DFE) of molecular complexes such as PPCs and PLCs. We used this procedure to calculate DFE values for 19 non-congeneric PPCs and hundreds of PLCs (eight different protein targets), and we achieved good to excellent correlations in comparison to experimental binding potencies.

**Figure 1:**
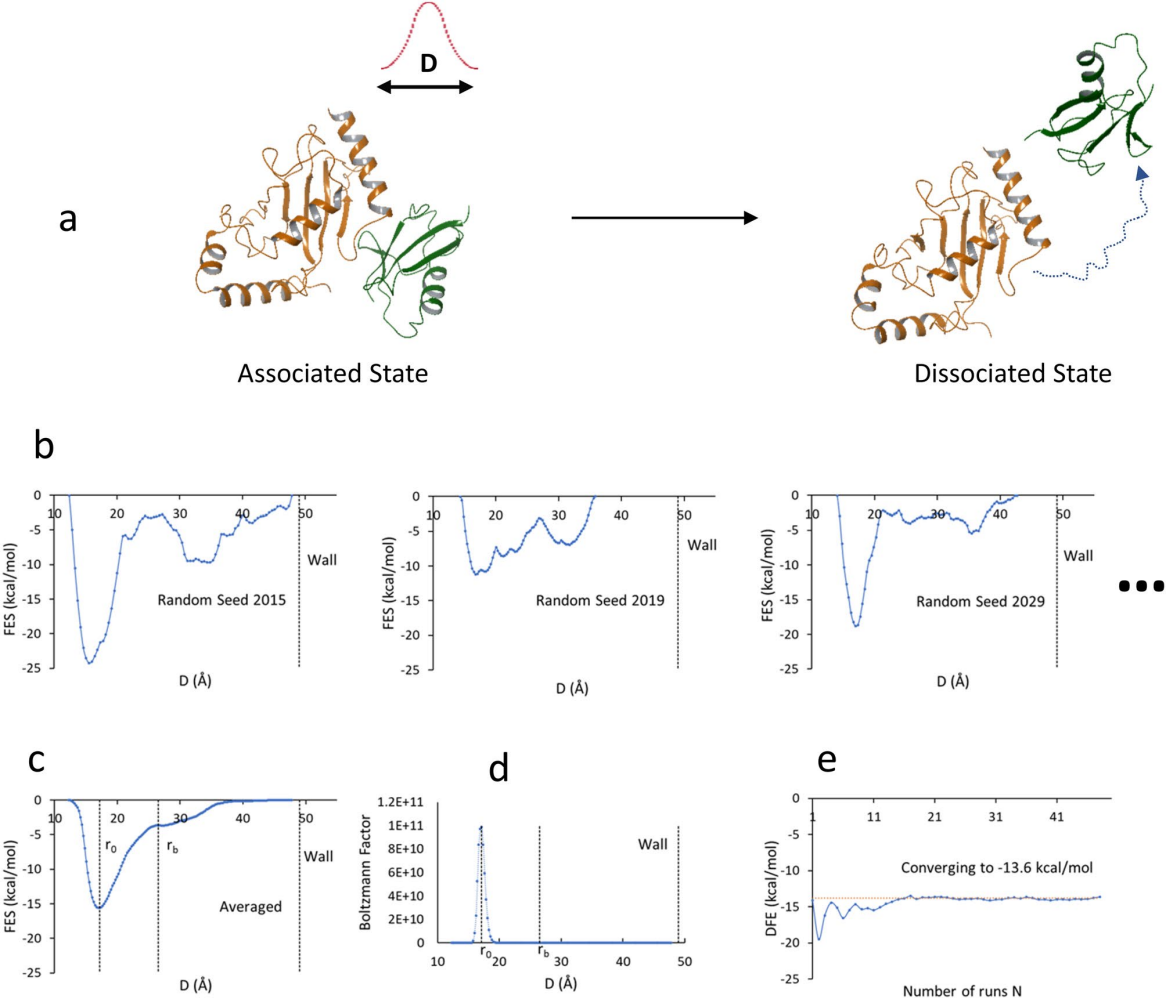
Concept and workflow of a DFE calculation. (**a**) One-way trip dissociation of a molecular complex by running metadynamics using an intermolecular distance D as CV. The dotted curve on the right panel is an illustrative trajectory of the centroid of the CV residue of protein B (green ribbon). (**b**) Primitive FES from a number of replicas of different random seeds, noting that a wall is placed in each run. (**c**) Average FES stemming from an ensemble of primitive FES, noting the position r_0_ of the bound state minimum and the boundary r_b_ separating the bound state region and the free state region. (**d**) Boltzmann factor as a function of D, narrowly concentrated around r_0_. (**e**) Convergence analysis: DFE is iteratively calculated as more and more replicas enter into averaging, showing that DFE converges when a sufficient number of replicas are averaged.

The key concepts of the calculation of DFE of a molecular complex are as follows. Assuming that the complex structure of two molecules is known and used as the starting point, a complex is dissociated by performing a standard metadynamics run with a user-defined distance between the two molecules used as the collective variable (CV). A Gaussian energy impulse centered at a running value of the CV is periodically added to the total potential energy of the system at every time interval along the progression of the simulation, which forces the CV to increase, leading to the dissociation of the molecules (see Fig. 1a). Unlike the usual metadynamics procedures which seek repeated back-and-forth sampling to achieve the convergence condition^16–18^, this procedure performs a number of one-way trip runs. In each run, the system undergoes dissociation and does not re-associate due to the immense space available to explore in the dissociated state. In such a run, how long the system stays in the bound (associated) state depends on the free energy well or the intrinsic stability of the complex, while how long it stays in the free (dissociated) state is arbitrary depending on the simulation time. As long as the system stays in the bound state, Gaussian energies will continuously be added into that region. When the accumulation of the Gaussian energies fills up the bound state well, the system exits from the bound state. Therefore, the accumulated Gaussian energies of the bound state provide the raw data from which we can calculate a free energy quantity, namely the DFE of the system. DFE can be understood as a measure of the degree of difficulty of pushing the system out of the bound state region. The Gaussian energies added into the free state do not contribute to this DFE and can be ignored. Giving that a single run inevitably includes errors associated with the random nature of the simulation, multiple runs are performed until the calculated DFE converges to a stable value. According to the metadynamics terminology, the distribution of the accumulated Gaussian energies (functions) is called the negative image of the free energy surface (FES, see Fig. 1b,c). Our procedure derives DFE from the FES profiles of the one-way trip runs.

We used the Desmond metadynamics module implemented in Maestro suite^19,20^ to do metadynamics runs and we wrote several scripts to perform job launches and post-run data processing including FES regeneration, averaging, DFE calculation and convergence analysis. However, this procedure can be readily reproduced based on other metadynamics programs.

## Results

### Summary of DFE procedure

The calculation of DFE of a complex is composed of the following steps. (1) A first set of independent metadynamics runs are launched. Each run shares the same starting point while using a different random seed, and typically proceeds for 10–40 ns. (2) After completion of these runs, the FES of each run (namely the primitive FES) is calculated (see Fig. 1b). (3) The averaged FES is calculated from an ensemble of primitive FES (see Fig. 1c). (4) DFE is calculated from an averaged FES. And (5) the convergence analysis is performed (see Fig. 1e). If convergence is not reached, another set of runs are launched and the previous steps are iterated; otherwise, the final DFE is obtained. After step 2, each run is examined to determine if it behaves as a one-way trip. A run that does not fit the one-way trip description is removed from the ensemble for averaging. A run that needs more simulation time to exit from BS is extended. This is called “the correction process” and will be detailed in the “Methods” section. The calculation of the primitive FES of an individual run is part of the standard metadynamics^16,19,20^. The calculation of DFE and the convergence analysis will be illustrated in more detail below.

The primitive FES from three of the runs of a PPC (PDB entry 3A4S) are given in Fig. 1b as examples. While their differences reflect the randomness of different runs, they all feature a major peak around the bound state region and a flatter variable curve in the free state region. The averaged FES from 50 runs for the same system is given in Fig. 1c, indicating a smoother surface, a clear bound state minimum centered at r_0_ and a boundary r_b_ between the bound state region and the free state region which is a maximum or saddle point.

### Calculation of DFE from averaged FES

Denoting the averaged FES using symbol g(D), a nominal partition function Q can be calculated using the following equation:

1 $$Q={(b-a)}^{-1}{\int}_{a}^{b}\text{exp}\left(-\frac{g(D)}{kT}\right)dD$$

where a and b are the beginning and end of the bound state region, respectively; k, T and D are the Boltzmann constant, temperature and CV distance, respectively.

It is important to point out that $\text{exp}\left(-\frac{g(D)}{kT}\right)$ decreases exponentially when g(D) increases. As shown in the example in Fig. 1d, this function is narrowly concentrated around r_0_. Only the region around r_0_ with energy higher than the minimum point by 4 kcal/mol or less has significant contributions to Q. Therefore, the integration can be done over the entire D region sampled, which would have no significant difference compared to integration over the bound state region. In other words, practically, *a* can be set to zero and *b* can be set to the distance where the wall is placed. (A wall is used to stop the monomers from moving too far from each other). One does not have to determine where r_b_ is to calculate Q.

DFE is calculated from the nominal partition function as,

2 $$DFE= -kT\text{ln}Q$$

### Convergence analysis based on DFE-N plot

DFE is calculated based on the averaged FES which depends on how many runs are used in the averaging process. To examine if DFE converges into a stable value for a given system, DFE is first calculated using only one run, and then recalculated iteratively by incorporating additional runs into the averaging. An example of a plot of DFE as a function of the number of runs (N) used in the averaging is shown in Fig. 1e. Large fluctuations in the calculated DFE are observed initially for small values of N and convergence to a stable value occurs as N is increased. When the fluctuations among the last five runs are smaller than 1 kcal/mol, the sampling is considered converged and the final DFE value is obtained.

### Calculation of DFE for a diverse set of PPCs

Nineteen PPCs were selected from the Protein–Protein Interaction Database which provides a collection of crystal structures of PPCs with the corresponding experimental binding affinities^21–27^. The details of the selection are given in the “Methods” section. The DFE procedure was applied to each complex.

**Figure 2:**
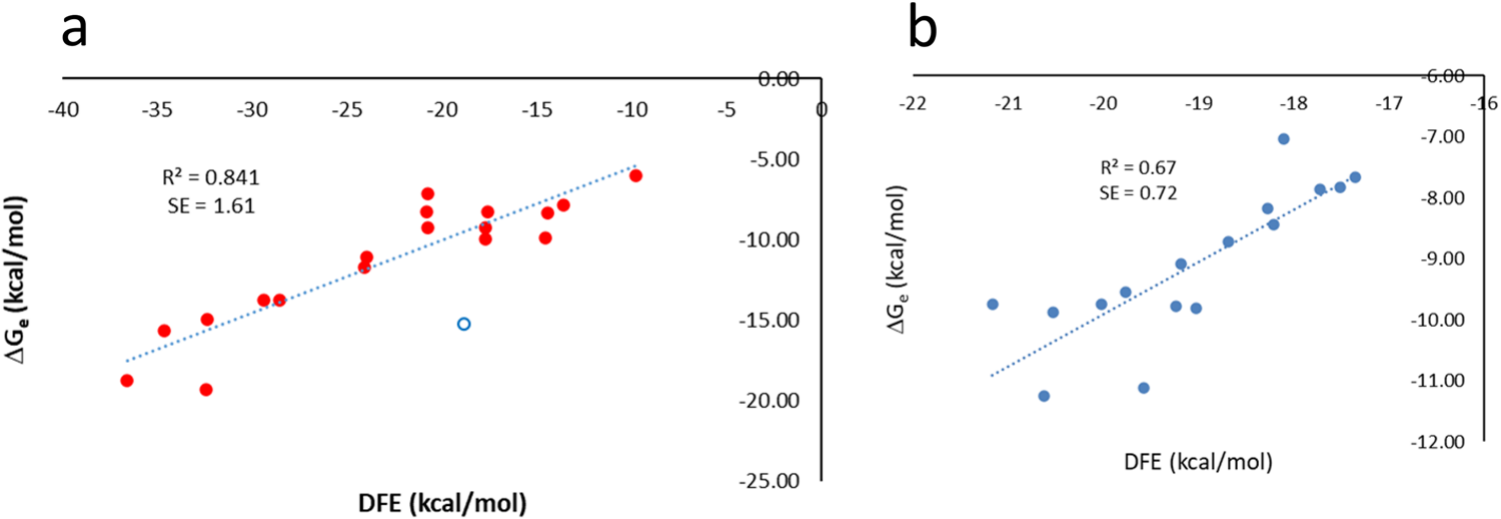
Correlation plots between calculated DFE and experimental binding free energies ∆G_e_. (**a**) Correlation plot for 19 PPCs. The open circle is an outlier (corresponding to complex 3SGB in Table 1). The indicated R^2^ and SE (Standard Error) correspond to the Least-Square-Fitting after excluding the outlier. (**b**) Correlation plot for PLCs for target CDK2.

The derived averaged FES and convergence plots for these PPCs are given in Supplementary Fig. S1. Columns 1–4 of Table 1 summarize each PPC’s PDB code, complex type, definition of CV and DFE value obtained, respectively. Columns 6–7 give the experimental binding free energy ∆G_e_ and dissociation constant K_d_ of each PPC. The plot of ∆G_e_ against calculated DFE for the 19 complexes (Fig. 2a) indicates a strong linear correlation between DFE and ∆G_e_, though one point (open circle) appears to be an outlier. If this point is excluded from the Least-Square-Fitting, the coefficient of determination R^2^ and the coefficient of correlation R are 0.84 and 0.92, respectively, indicating a very high correlation. The relationship between DFE and ∆G_e_ from the Least-Square-Fitting was as follows, with a standard error of 1.61 kcal/mol.

**Table 1:** Calculation of DFE of 19 non-congeneric PPCs to compare with experimental binding free energies.

PDB code	PPI type	CV^a^	DFE (kcal/mol)	∆G_c_^b^ (kcal/mol)	∆G_e_^c^ (kcal/mol)	K_d_^d^ (M)
1EMV	IM9 immunity protein–Colicin E9 nuclease	V37^A^–F86^B^	− 32.42 ± 0.16	− 15.65	− 19.32	2.4 × 10^–14^
2PTC	Trypsin–BPTI	D194^E^–K50^I^	− 36.59 ± 0.27	− 17.53	− 18.75	6 × 10^–14^
1BVN	α-Amylase–Tendamistat	D300^P^–A823^T^	− 34.67 ± 0.07	− 16.66	− 15.65	9.2 × 10^–12^
1R0R	A serine protease–OMTKY3	L126^E^–T17^I^	− 32.38 ± 0.13	− 15.63	− 14.94	2.94 × 10^–11^
1ACB	Chymotrypsin–Eglin C	S214^E^–L45^I^	− 29.38 ± 0.2	− 14.27	− 13.76	2 × 10^–10^
1AY7	Rnase SA–Barstar	T82^A^–L20^B^	− 28.54 ± 0.18	− 13.9	− 13.76	2 × 10^–10^
2UUY	Trypsin–Tryptase inhibitor	D196^E^–K39^I^	− 24.11 ± 0.32	− 11.9	− 11.7	5.6 × 10^–9^
1KAC	Adenovirus protein–Human receptor	L430^A^–W59^B^	− 23.99 ± 0.16	− 11.84	− 11.11	1.48 × 10^–8^
3BZD	TCR Vβ8.2–Enterotoxin C-3	F75^A^–M24^B^	− 17.73 ± 0.23	− 9.02	− 9.95	9.6 × 10^–8^
2C0L	PEX5–SCP2	S612^A^–L31^B^	− 14.56 ± 0.15	− 7.59	− 9.88	1.09 × 10^–7^
1KTZ	TGFβ–TGFβ receptor	V33^A^–T51^B^	− 20.78 ± 0.05	− 10.39	− 9.27	2.9 × 10^–7^
3LVK	Cys desulfurase–Sulfurtransferase	D52^A^–R27^B^	− 17.72 ± 0.17	− 9.01	− 9.25	3 × 10^–7^
1FFW	Chemotaxis protein CheY–Chemotaxis protein CheA	L84^A^–L212^B^	− 14.46 ± 0.07	− 7.54	− 8.33	1.35 × 10^–6^
3F1P	HIF2A–ARNT	V340^A^–I458^B^	− 20.83 ± 0.47	− 10.42	− 8.3	1.4 × 10^–6^
1US7	HSP90–P50	F104^A^–L205^B^	− 17.59 ± 0.16	− 8.96	− 8.28	1.46 × 10^–6^
3A4S	UBC9–SLD2	I45^B^–I408^C^	− 13.61 ± 0.17	− 7.16	− 7.87	2.81 × 10^–6^
1QA9	CD2–CD58	K91^A^–D33^B^	− 20.74 ± 0.12	− 10.38	− 7.16	9 × 10^–6^
2OOB	CBL-B–Ubiquitin	L69^B^–A937^A^	− 9.81 ± 0.15	− 5.44	− 5.99	6 × 10^–5^
3SGB	Proteinase B–OMTKY3	L18^I^–S195^E^	− 18.84 ± 0.03	− 9.52	− 15.24	1.79 × 10^–11^

^a^A CV was defined by the distance between the centroid of the backbone heavy atoms of a residue in protein 1 and the centroid of the backbone heavy atoms of a residue in protein 2. For 2PTC, 2C0L, 3LVK, 3F1P and 2OOB, the C^β^ was also included in the definition of the corresponding centroid.

^b^Calculated binding free energy by introducing DFE into Eq. (3) in the text.

^c^Experimental binding free energy calculated using ∆G_e_ = kT ln(K_d_) at T of 310 K.

^d^Experimental binding constant K_d_ from ref.^27^.

3 $$\Delta {G}_{e}=0.4512 DFE-1.02$$

If the outlier is not excluded, R^2^, R and the standard error change to 0.74, 0.86 and 2.06, respectively, which still indicates a high correlation.

The results indicate that DFE is linearly correlated with ∆G_e_, but the individual DFE values differ from the corresponding individual ∆G_e_ values. This is consistent with the concept that DFE is not the binding free energy by definition, but it is closely coupled with it. The major difference between the concept of DFE and that of binding free energy is that DFE quantifies the work required to dissociate a complex while binding free energy is the free energy difference between the bound and free states. DFE does not require the calculation of the free state explicitly, neither does it require the calculation of the contribution of the standard state concentration^5,28^. The ignored terms in DFE may be mostly the constant terms of the binding free energy. More analysis about what are missing in DFE is given in the “Discussion” section.

The above relationship between ∆G_e_ and DFE was used to convert the DFE values into calculated binding free energies ∆G_c_ (Column 5, Table 1). ∆G_c_ can be compared with ∆G_e_ individually. The errors given in the DFE column of Table 1 are due to the sampling completeness. The differences between ∆G_c_ and ∆G_e_ are typically larger than these errors because they include everything that could cause discrepancies between calculations and experimental measurements including theoretical assumptions, force-field errors and experimental errors.

### Comparison with and without the correction process

The above results were obtained after applying the correction process. Table 2 gives a comparison of DFE and ∆G_c_ with and without the correction process (columns 5–8) as well as the total number of runs, chemical time in each run before any extension, and corrections made if any, for each PPC, respectively. Overall, about 13% of the runs could not be considered as completed one-way trips. These runs were either extended to longer simulation times or removed from the calculations according to the process detailed in the “Methods” section. Interestingly, the correction process did not change ∆G_c_ significantly for any of the complexes; the changes due to the correction are either close to or smaller than 0.5 kcal/mol. In terms of DFE, only three of the 19 complexes showed a difference more than 1 kcal/mol (1.2, 1.3 and 2.2) before and after the correction. The correlation indexes R^2^ (0.84) and R (0.91) without the correction remained practically the same as those with the correction.

**Table 2:** Comparison of calculated DFE and binding free energies ∆G_c_ before and after the correction.

PDB code	N^a^	t^b^ (ns)	Applied corrections	DFE^c^ (kcal/mol)	DFE^nc d^ (kcal/mol)	∆G_c_^e^ (kcal/mol)	∆G_c_^nc f^ (kcal/mol)
1EMV	50	40	No corrections	− 32.42	− 32.42	− 15.65	− 15.57
2PTC	50	20	18 runs extended to 30 ns; 2 runs to 40 ns	− 36.59	− 35.95	− 17.53	− 17.43
1BVN	50	40	No corrections	− 34.67	− 34.67	− 16.66	− 16.57
1R0R	50	20	No corrections	− 32.38	− 32.38	− 15.63	− 15.55
1ACB	50	30	No corrections	− 29.38	− 29.38	− 14.27	− 14.22
1AY7	60	40	13 runs with Invasions removed	− 28.54	− 30.76	− 13.9	− 14.36
2UUY	50	20	4 runs extended to 30 ns	− 24.11	− 24.14	− 11.9	− 11.88
1KAC	50	30	No corrections	− 23.99	− 23.99	− 11.84	− 11.82
3BZD	50	20	3 runs with Invasions removed	− 17.73	− 17.77	− 9.02	− 9.04
2C0L	50	20	13 runs with repeated sampling removed	− 14.56	− 15.9	− 7.59	− 7.61
1KTZ	50	20	8 runs extended to 30 ns	− 20.78	− 20.79	− 10.39	− 10.39
3LVK	50	20	3 runs with repeated sampling removed; 11 runs extended to 30 ns; 2 runs to 40 ns	− 17.72	− 16.89	− 9.01	− 9.02
1FFW	50	10	11 runs with repeated sampling removed; 6 runs extended to 20 ns	− 14.46	− 13.24	− 7.54	− 7.03
3F1P	50	30	7 runs with repeated sampling removed; 2 runs extended to 40 ns	− 20.83	− 20.31	− 10.42	− 10.41
1US7	50	10	No corrections	− 17.59	− 17.59	− 8.96	− 8.96
3A4S	50	10	2 runs with Invasions removed; 17 runs extended 20 ns	− 13.61	− 14.42	− 7.16	− 7.55
1QA9	50	20	No Corrections	− 20.74	− 20.74	− 10.38	− 10.37
2OOB	50	10	2 runs with repeated sampling removed; 5 runs extended to 20 ns	− 9.81	− 9.54	− 5.44	− 5.5
3SGB	50	40	No correction	− 18.84	− 18.84	− 9.52	− 8.96

^a^The number of runs.

^b^Chemical time of a run before the correction process.

^c^DFE after the correction process.

^d^DFE before the correction process.

^e^Calculated binding free energy after the correction process.

^f^Calculated binding free energy before the correction process.

### Calculation of DFE for eight protein targets with different sets of ligands

To investigate the performance of the DFE procedure in predicting protein–ligand binding free energies, we used a published dataset of PLC structures with experimental binding affinities, which had previously been used to benchmark the FEP method^10^.

The derived averaged FES and convergence plots of all the PLCs are given in Supplementary Figs. S2 and S3. Columns 1–5 in Table 3 give the protein targets, number of ligands for each target, CV definition, number of runs and chemical time in each run, respectively. The obtained DFE were compared with experimental ∆G_e_ for each target separately, with the Least-Square-Fitting analysis. The derived R^2^ and standard error for each target are included in Columns 6–7 of Table 3. R^2^ varied from 0.67 for CDK2 to 0.32 for BACE. R^2^ for Thrombin was exceptionally low (0.01), however, exclusion of two outliers brought R^2^ to 0.62. The average R^2^ across all the eight targets was 0.45 without excluding any points and 0.53 after excluding the two outliers in Thrombin data. Interestingly, the standard error did not follow the trend of R^2^, but distributed flatly between 0.57 and 1.09 kcal/mol. Thrombin had the smallest standard error. These observations suggest that the prediction errors for different targets are similar and reasonably small, and that the variations of R^2^ among different targets are mainly due to the different binding free energy spans of the ligand sets. For example, the span for CDK2 is 4.2 kcal/mol while the span for Thrombin is 1.7, causing a higher R^2^ for the former than for the latter. Likewise, the R^2^ for these PLCs are generally smaller than the R^2^ for the PPCs because the latter has a much larger binding free energy span (13 kcal/mol).

**Table 3:** R^2^, standard errors (SE) and relationships between DFE and experimental binding free energies ∆G_e_ for 8 protein targets with different sets of ligands, as well as the simulation conditions.

Target	Number of ligands	CV^a^	N^b^	t^c^ (ns)	R^2^	SE (kcal/mol)	Relationship
CDK2	16	F80	40	20	0.67	0.72	∆G_e_ = 0.858DFE + 7.26
TYK2	16	M978	50	10	0.66	0.79	∆G_e_ = 0.602DFE − 0.85
P38α	34	L75	40	20	0.6	0.65	∆G_e_ = 0.39DFE − 3.33
JNK1	21	L110	27	10	0.51	0.62	∆G_e_ = 0.559DFE − 2.86
MCL1	42	L290	30	10	0.48	0.78	∆G_e_ = 0.642DFE − 1.96
PTP1B	23	R221	40	15	0.35	1.09	∆G_e_ = 0.628DFE + 4.19
BACE	36	W176	40	20	0.32	0.65	∆G_e_ = 0.268DFE − 4.18
Thrombin^d^	11	S214	50	10	0.01 (0.62)	0.57 (0.36)	(∆G_e_ = 0.793DFE + 0.37)
Average^d^					0.45 (0.53)	0.73 (0.71)	

^a^A CV was defined by the distance between the centroid of the backbone heavy atoms of a residue of the corresponding protein and the centroid of the heavy atoms of the corresponding whole ligand, except that, for TYK2 and Thrombin, the C^β^ was also included in the definition of the corresponding centroid; and for CDK2, P38α and BACE, the heavy atoms of a whole residue were used.

^b^The number of runs performed for each ligand.

^c^Chemical time of a run.

^d^The numbers in the parentheses correspond to the results after exclusion of two outlier points in the Thrombin data set.

The linear relationship between DFE and ∆G_e_ derived from Least-Square-Fitting for each target is given in Column 8, Table 3. The correlation plots are given in Fig. 2b for CDK2 and Supplementary Fig. S4 for all the targets.

The overall predictive power of the DFE method was similar to that of the FEP+ method^10^ for these same sets of PLCs. A comparison of the R^2^ and standard error between the two methods in terms of predicting experimental binding free energies is summarized in Supplementary Table S1 and Figs. S4 and S5.

### Definition of appropriate CVs and assessment of variability due to the different CVs

The intermolecular distance used as the CV in the calculation of DFE of a complex needs to be defined such that its forced increase directly leads to the dissociation of the complex without significant conformational changes unrelated to the dissociation. Although it is difficult to differentiate which conformational changes are related to the dissociation and which are not, we took the following precautions in the choice of CVs: (1) we avoided using any residues from flexible loops to define a CV because the Gaussian forces acting upon such a CV could cause conformational changes of the corresponding loops which might not originate from the dissociation process. (2) The CV for a PPC was defined as the distance between the centroids of the backbone heavy atoms of a pair of residues, each from different proteins in the complex. The CV for a PLC was defined as the distance between the centroid of the heavy atoms of a residue of the protein and the centroid of the heavy atoms of the whole ligand in the complex. And (3) the protein residues used to define a CV were tightly packed within the respective proteins and were located within either an α-helix or a β-sheet of multiple strands when possible.

We used one PPC (3A4S) to explore the effect of the change of CV definition on DFE, in which we tried seven different CVs by changing the residues defining the CV. For each of the CVs, DFE was respectively calculated with and without the correction process (see Supplementary Table S2). We found that the standard deviation of DFE stemming from the changes of CV definition was about 1.08 kcal/mol without the correction process and 1.03 with the correction process. The standard error of the mean was 0.41 and 0.39 kcal/mol, respectively. The maximum difference among the set of DFE values was about 2.73 without the correction and 2.96 with the correction. Substituting these values into Eq. (3), the estimated uncertainty in the predicted ∆G_c_ due to the different choices of CV was less than 1.3 kcal/mol, or 0.5 based on the standard deviation of DFE. Given that the standard error of Eq. (3) was about 1.6 kcal/mol, the uncertainty due to the different choices of CV would not alter the described quality of correlation largely, and it might be one of the sources of error already included in the standard error of Eq. (3).

## Discussion

It is striking that the calculated DFE generally showed strong correlations with the experimental binding free energies for such a variety of systems. We offer the following thoughts concerning what factors and subprocesses of an association/dissociation event are included and what are absent in a DFE calculation. (1) Conceptually, the DFE of a complex is derived from the sampling of the time period of the entire bound state up to a point where the system exits from the bound state well. (2) The factors captured include at least the interactions between and within the solute molecules, effects stemming from the thermal motions, conformational changes within the sampled period and solvent effects. And (3) the period after the dissociation is not considered, in which the solutes undergo free movements and isolated conformational variations as well as much weaker nonspecific interactions. The free movements of the solutes in the free state are independent of the solute molecular interactions, contributing a constant term to the binding free energy. The nonspecific interactions of the system after its exit out of the bound state are solute-dependent but may be much weaker relative to the bound state interactions and the entropic term of the free movements of the free state. The role of the conformational changes or dynamics of solutes in the free state (before one solute finds an entrance into another) is not captured in DFE and may be significant for some systems. More studies may be useful to clarify this point in the future. However, the role of the conformational changes or dynamics for the period between the entrance and the “endpoint” of the bound state is accounted for in the DFE procedure.

Although the DFE procedure samples the free energy surface based on the dissociation events, the free energy surface obtained would be the same as that obtained from the association events for the bound state region. This is because binding is a reversible process. An event that travels from the free state to the bound state (namely association event) would sample the same bound state free energy well as an event from the opposite direction (dissociation event). This is true for both real and simulated events. Based on this reasoning, the calculated free energy should have captured the effects of part of both on- and off- processes of binding, the part that covers the bound state phase.

In general, the averaged FES has a primary minimum corresponding to the bound state significantly deeper than other regions along the CV. As a result, the derived DFE primarily depends on the bottom of the minimum (within 4 kcal/mol from the minimum point). The sampling beyond this bottom region does not contribute to the DFE significantly. In the other words, in addition to the free state region being unimportant for DFE, the transition state and bound state regions beyond the bottom of the minimum are also unimportant. This implies that after the system moves away from the bottom region, even though the molecules may retain much of the native interactions and/or gain some nonnative interactions, the simulation of these regions does not contribute to the DFE significantly. This is counterintuitive but true, and it is one of the reasons for the robustness of the DFE calculation. However, this does not mean that some parts of the native interactions are not needed for DFE. Every part of the native interactions contributes to the free energy values of the bottom region of the minimum and thus contributes to the DFE.

Some comparisons between our DFE method and the traditional potential of mean force methods^4–6^ and between DFE and other metadynamics methods^7–9^ are given as follows. (1) In the potential of mean force methods, a complex is dissociated by forcing one of the binding partners to move relative to the other along a user-defined path. Such a steered movement is achieved by dividing the path into n windows and using restraining forces to gradually move the system from one window to the next. In comparison, a DFE run does not require a user-defined path: the system moves in an unrestrained molecular dynamics simulation governed by the total energy which is the sum of the normal molecular energy and the added Gaussian functions. Gaussians are periodically added to wherever the system is in an unrestrained simulation rather than to pre-defined locations. The system is driven out of the bound state well by the accumulation of the Gaussian energies via any pathways that it can find at random in the simulation. (2) Unlike the potential of mean force methods, a DFE simulation does not require any restraints or constraints on any translational, rotational or conformational variables of the molecules. (3) Unlike other metadynamics methods, a DFE simulation does not use comprehensive collective variables but uses a single physical distance as the collective variable. And (4) the DFE procedure achieves convergence despite the above three simplifications because it uses one-way trip simulations in which the dissociated partners are not required to re-associate, and it reaches the convergence by averaging multiple independent runs. In the traditional methods, the dissociated partners are required to re-associate (backward trips) for convergence, which poses a problem that the partners cannot find the original native interaction mode if their orientations and conformations are not restrained. Thus, there is a need of imposing system-dependent restraints or constraints and using comprehensive collective variables in the traditional methods. In contrast, the DFE method is free of these complications and can be performed much more easily.

One drawback of this method is that it does not predict binding modes. One remedy is to combine DFE with docking using DFE to select binding modes and generate more accurate estimates of binding free energies.

The recent success of prediction of protein structures of almost the entire human proteome with high accuracy with AI-driven AlphaFold^29,30^ methodology has removed a major obstacle hampering structure-based drug design. The combined use of DFE with artificial intelligence^31^, docking^32,33^ and de novo design^34–36^ could overcome the remaining barriers and make rational drug design a widely viable and reliable approach. Several important issues such as hit identification, potency, selectivity and off-target toxicity can be addressed using this type of structure-based approach. This applies both for small molecule drugs and biologic drugs. And finally, DFE can also be used to study protein–protein interactions of trimer complexes in the design of PROTACS^37–41^.

## Methods

### Major steps of a DFE calculation

The calculation of DFE of a complex consists in a multi-step procedure by employing the Desmond module implemented in Maestro Drug Discovery Suite^42^ (version 2019-3 with OPLS3e force-field^43^) to generate raw data, and by processing the raw data to obtain DFE using our own scripts. It is composed of the following steps.

**Figure 3:**
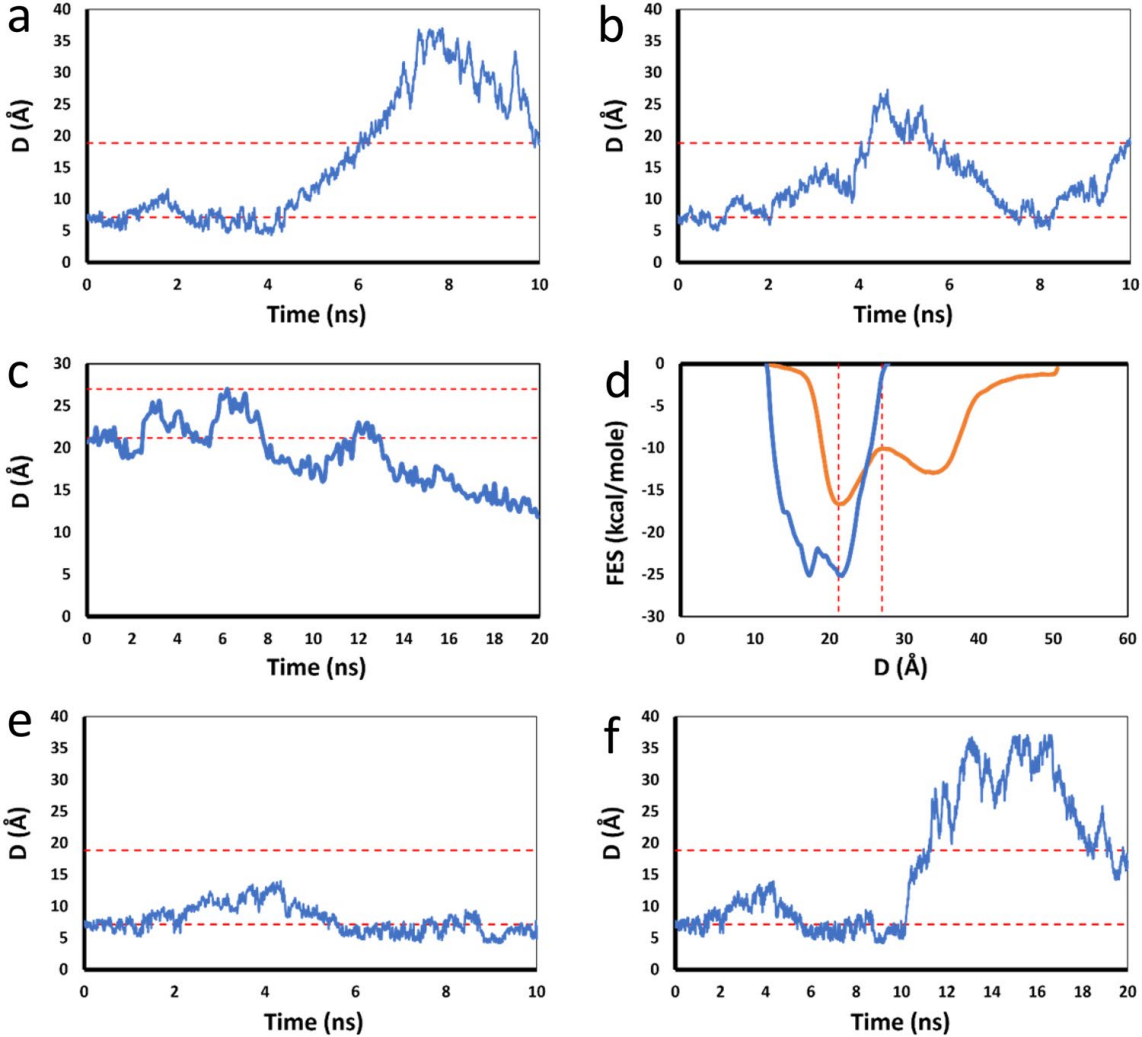
Examination of each run against one-way trip description. (**a**) D versus Time plot of a one-way trip run in which the sampling initially stays around r_0_ (lower-level dotted red line), and then goes up passing through r_b_ (higher-level dotted red line) without returning back to the level around r_0_. (**b**) D versus Time plot of a multi-trip run in which the sampling goes up passing through r_b_ and then returns back to the original level around r_0_. (**c**) D versus Time plot of a run where D goes toward opposite direction of dissociation signaling invasions, conformational changes or other movements unrelated to the dissociation. (**d**) Average FES (orange curve) and the primitive FES (blue curve) corresponding to the run in panel (**c**). The primitive FES of this run invades the “inner” region, left to the left wall of the primary minimum of the average FES. (**e**) D versus Time plot of a run that stays in the bound state because the simulation time is not long enough. (**f**) D versus Time plot after the run in panel e is extended to longer simulation time, showing a one-way trip behavior.

1. Protein preparation. The crystal structure is imported into Maestro GUI. The Protein Preparation Wizard panel^44^ is used to add hydrogen atoms, patch end groups, add missing side chains and missing loops, assign protonation states of histidine, aspartate and glutamate at pH 7.0^45^ and optimize the polar hydrogen orientations in crystal water molecules and proteins.
2. Simulation system setup. The complex is solvated in a cubic solvent box of which the size is set to be about the sum of the largest dimension of the complex and 35 Å. The solvents are composed of water molecules, sodium ions and chloride ions with a salt concentration of 0.15 M. Any potential ligands are checked with Force Field Builder module. If dihedral angles with suboptimal parameters exist, the corresponding parameters will be generated using this module which fits the force-field energy against the quantum mechanical energy for the related fragments.
3. Preparation of metadynamics input files. The solvated complex is loaded into the Metadynamics panel. The CV distance is defined. The Gaussian width and height are set to 0.05 Å and 0.01 kcal/mol, respectively. A wall at the CV value equal to the sum of the largest dimension of the complex and 30 Å is set to prevent the CV from going too far. The time interval between which a Gaussian is injected is set to 0.09 picoseconds (ps). The simulation temperature and pressure are set to 310 K and 1.01325 bar, respectively. The simulation time is set to the desired time (usually 10–40 ns). This step generates three input files (cfg, msj and cms), a command file (sh) and a force-field directory opls_dir.
4. Generation and submission of multi-replica jobs. This step is done using one of our scripts. The initial input directory and the input files therein are replicated into multiple replicas which differ from each other only by the random seeds used. A typical scenario is 50 replicas with random seed changing from 2007 to 2056. Each replica is launched into a different instance of type p3.xlarge in an AWS virtual cluster, and each replica employs one GPU of type V100 and one CPU.
5. Multisim^19,20^ run of each replica. Each replica run undergoes multiple stages as instructed in the msj file as follows. (a) Brownian dynamics of NVT ensemble: temperature 10 K, small timesteps, restraints on solute heavy atoms and 100 ps. (b) Berendsen dynamics of NVT ensemble: temperature 10 K, small timesteps, restraints on solute heavy atoms and 12 ps. (c) Berendsen dynamics of NPT ensemble: temperature 10 K, restraints on solute heavy atoms and 12 ps. (d) Berendsen dynamics of NPT ensemble: temperature 310 K, restraints on solute heavy atoms and 12 ps. (e) Berendsen dynamics of NPT ensemble: temperature 310 K, no restraints and 500 ps. (f) Production. Martyna-Tobias-Klein dynamics of NPT ensemble with metadynamics is performed at a temperature of 310 K without restraints up to the targeted simulation time. This generates a kerseq file recording all the Gaussian functions infused into the system along the simulation time, the trajectory file and other output files.
6. Calculation of primitive FES from each replica run. The expression of the Gaussian energy function G(D) that is added to the system at a time point t is
  4 $$G\left(D\right)=h \text{exp}\left(-\frac{{(D-d(t))}^{2}}{2{\sigma }^{2}}\right)$$
  where d(t) is the value of the CV at time t serving as the center of the Gaussian function and D is the CV serving as the variable of the Gaussian function; h and σ are the user-defined Gaussian height and width, respectively^16^. D is a spatial variable that covers the full range of the CV variations.
  The free energy value at a CV point is the negativity of the sum of all the Gaussian energies at that point. The corresponding distribution of the free energy versus the CV is the so-called FES. Although the module automatically generates a FES file at the end of each run, a new FES is generated by dividing the sampled region of CV into 80 equal intervals and calculating the free energy at each division point by summing up the values of all the Gaussian energies at that point, based on the Gaussian functions recorded in the kerseq file. The resulted FES file contains an 80 × 2 table in which each row is composed of a CV value and the corresponding free energy value, and there are 80 such rows.
7. Calculation of average FES. This step is done using one of our scripts with an algorithm as follows. The primitive FES files of all the runs are scanned to find the lowest and highest values of CV that have been visited. The segment of CV between the lowest value and the highest value is divided into equal intervals with the interval size equal to the smallest of the different interval sizes of different primitive FES. The free energy value at each division point (based on this new division) for each primitive FES is calculated based on the two closest points in the previous primitive FES file using a linear interpolation scheme. Assuming that, for a division point at CV value D in the new division, the closest points to D in the old primitive FES file correspond to D_1_ (D_1_ < D) and D_2_ (D_2_ > D) and the free energy values at D_1_ and D_2_ are g_1_ and g_2_, respectively, the free energy value g at D is as below.
  5 $$g= {g}_{1}+(D- {D}_{1})\frac{{(g}_{2}- {g}_{1})}{({D}_{2}-{D}_{1})}$$
  After the recalculation of each primitive FES for the common division scale above, the average free energy value at each division point of CV is calculated by averaging the free energy values from all the primitive FES of replicas.
8. Examination of each run against one-way trip criteria and the correction process. The plot of CV versus simulation time and the plot of primitive FES are plotted for each run using one of our own scripts. And the average FES is overlaid to the primitive FES in the latter plot. The position of the native minimum r_0_ and the boundary r_b_ between the bound state and the free state are determined based on the average FES. A one-way trip run is a run that the CV travels from the bound state region (< r_b_) to the region outside of it (> r_b_), and it does not come back as reflected in the CV versus time plot (see Fig. 3a). If it comes back to the bound state region after it has exited from it, it is considered as a multiple-way trip run (see Fig. 3b), which should be rejected from the ensemble for the averaging of FES. If the CV in a run does not exit from the bound state but it goes smaller and smaller to the extent less than r_0_ by more than 4 Å (see Fig. 3c), and its primitive FES shows significant invasion to the region left to the inner wall of the primary minimum of the average FES (see Fig. 3d), it is a run that leads to the continuous decrease of the CV distance via conformational changes and other movements unrelated to the dissociation process, and it should be rejected from the averaging. If a run has not exited from the bound state but it does not belong to the previous category (see Fig. 3e), this run should be extended to longer simulation time so that it can exit from the bound state (see Fig. 3f). If the last case fits to the one-way trip description after the extension, it is used in the averaging; otherwise, it will be discarded too. The whole process above is called the correction process. Interestingly, this correction process did not have much impact to the final DFE results in our studies, because most runs belonged to the one-way trip category. This process is currently manual, but it can be automated with a script if it is necessary in the future.
9. Calculation of DFE. The average FES is used in Eq. (1) to calculate a nominal partition function Q, and DFE is calculated from Q based on Eq. (2), using one of our scripts.
10. Convergence analysis. After the completion of the first set of replicas, DFE is first calculated using the first replica only, and then recalculated repeatedly by adding more and more replicas into the averaging. DFE is plotted as a function of the number N of the replicas used in the averaging. If this plot shows that DFE fluctuations become smaller and smaller when N becomes greater, the sampling is trending toward convergence. When the last five points in this plot have fluctuations less than 1 kcal/mol, the convergence is considered to have reached and the sampling stops. Otherwise, another set of replicas are launched and the previous steps are iterated until the convergence is reached. (In our applications, the first set had 30–50 replicas depending on the systems and the convergence was reached without the need of a second set for any of the systems).

### Approximate speed

The time of calculation of DFE of a complex is dominated by the metadynamics simulation time, since the time to run the scripts for post-run data processing is insignificant. Assuming that one deploys sufficient instances to allow all the runs of a complex to go in parallel, and that each run has a length of about 10–40 ns, the whole process takes about 1–4 h on p3.xlarge/V100 instances (excluding the time of the manually-performed correction process). In the calculations of PLCs, the computer time of the DFE was about the same as that of the FEP+^10^ on a per ligand basis.

### Crystal structures of PPCs and PLCs and experimental binding affinities

A small subset of PPCs were selected from the Protein–Protein Interaction Affinity Database 2.0 which contains 179 high quality PPC crystal structures with the corresponding experimental binding constants, the so-called “affinity benchmark” that the researchers had prepared to validate various protein–protein docking programs^21–27^. We carefully analyzed the full set and removed the complexes where the protein–protein interfaces met the following conditions: (1) consisted of more than two proteins, (2) were proximal to the truncated termini of protein constructs used in the structures, (3) involved coordinated metal ions, and (4) were close to missing loops of the proteins. In addition, the complexes that contain ATP or GTP analogs were also removed since the experimental interaction geometries around these groups were usually difficult to be maintained by constraint-free simulations due to the existence of coordinate bonds and strong charge-charge interactions. From the remaining structures, we randomly selected 19 complexes. These complexes spanned the full experimental affinity range from micromolar to femtomolar K_d_ about uniformly and were structurally diverse in size and nature of interactions. Most binding interfaces consisted of a mixture of hydrophobic interactions, hydrogen bonds and salt bridges. Our selected complexes included the ones interacting mostly through the helical packing or beta-sheet interfaces, as well as the ones interacting via a mixture of different secondary structure elements. Thus, we selected a reasonably diverse subset of protein complexes to test the performance of DFE method. The PLC structures and the binding data were taken from reference^10^ without exception.

## Supplementary Information

Supplementary Information.

## Data Availability

The Maestro and Desmond input files for each system modeled in this manuscript have been placed in a compressed folder named input_files available from Supplementary Data section.
