# Supplementary material for: A highly accurate metadynamics-based Dissociation Free Energy method to calculate protein–protein and protein–ligand binding potencies
Source: Sci Rep. 2022 Feb 7;12:2024. doi: 10.1038/s41598-022-05875-8 (PMC8821539; doi:10.1038/s41598-022-05875-8)
Supplement: Supplementary file 1 — Supplementary Information. [file 41598_2022_5875_MOESM1_ESM.pdf]

Supplementary Information

A highly accurate metadynamics-based Dissociation Free Energy method to calculate protein-protein and protein-ligand binding potencies

Jing Wang[1,*], Alexey Ishchenko[1], Wei Zhang[1], Asghar Razavi[1] and David Langley[1]

Supplementary Table S1. Comparison of DFE method and FEP+ method in terms of R^2 and SE (standard error) in Least-Square-Fitting of calculated free energies into experimental binding free energies.

Target	DFE-ΔG_e R^2 [a]	DFE-ΔG_e SE (kcal/mol)[a]	ΔG_{FEP+}-ΔG_e R^2 [b]	ΔG_{FEP+}-ΔG_e SE (kcal/mol)[b]
CDK2	0.67	0.72	0.23	1.11
TYK2	0.66	0.79	0.79	0.61
P38α	0.6	0.65	0.43	0.78
JNK1	0.51	0.62	0.72	0.47
MCL1	0.48	0.78	0.6	0.69
PTP1B	0.35	1.09	0.64	0.8
BACE	0.32	0.65	0.61	0.49
Thrombin[c]	0.01 (0.62)	0.57 (0.36)	0.5	0.41
Average[c]	0.45 (0.53)	0.73 (0.71)	0.56	0.67

[a]Derived by Least-Square-Fitting of calculated DFE to experimental ΔG_e. Same numbers have been reported in Table 3.

[b]Derived by Least-Square-Fitting of ΔG_{FEP+} to ΔG_e, both taken from Wang, L. et al. *J. Am. Chem. Soc*. **137**, 2695−2703 (2015).

[c]The numbers in the parentheses correspond to the results after exclusion of two outlier points in the Thrombin data set.

Supplementary Table S2. Calculated DFE of PPC 3A4S for different definitions of CV before and after the correction process.

CV[a]	DFE without correction (kcal/mol)	DFE with correction (kcal/mol)
$I45^{B}$-$V345^{C}$	-13.8	-13.04
$I45^{B}$-$I408^{C}$	-14.42	-13.61
$I112^{B}$-$V345^{C}$	-14.46	-14.63
$L113^{B}$-$V345^{C}$	-14.72	-14.16
$L113^{B}$-$I408^{C}$	-16.14	-15.16
$W16^{B}$-$V345^{C}$	-16.17	-15.28
$W16^{B}$-$L407^{C}$	-16.53	-16
Standard Deviation[b]	1.08	1.03
SEM[c]	0.41	0.39

[a]A CV is defined as the distance between the centroid of the backbone heavy atoms of a residue from protein B and the centroid of the backbone heavy atoms of a residue from protein C.

[b]Standard deviation among the set of DFE values in the rows above.

[c]Standard error of the mean among the set of DFE values in the rows above.

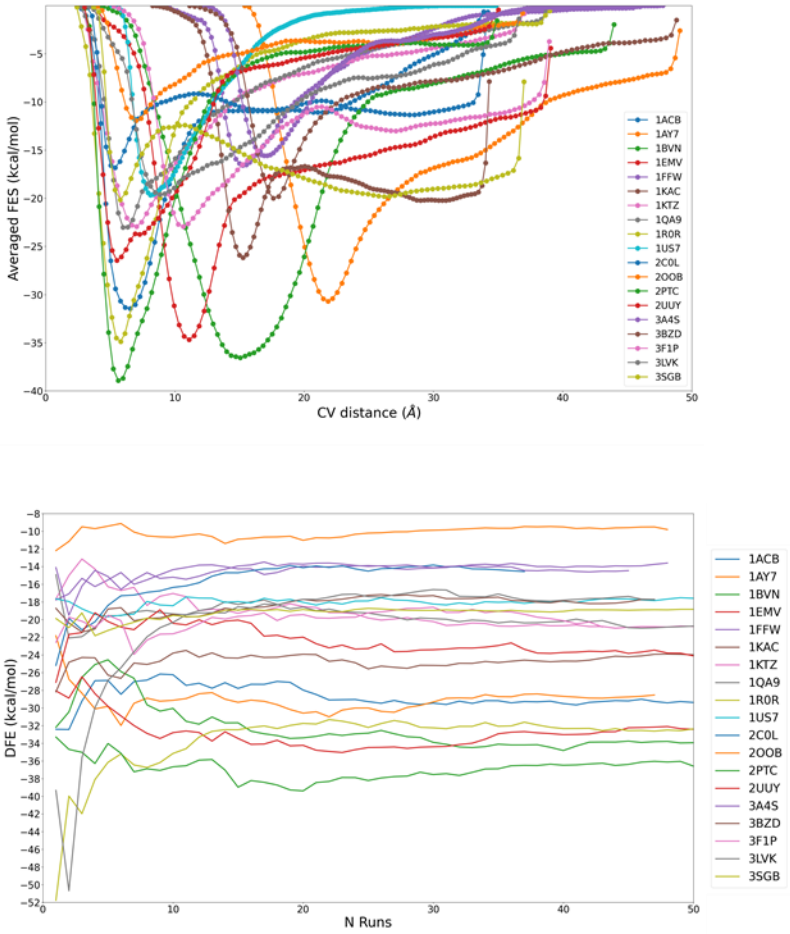

Supplementary Figure S1. Averaged FES (upper panel) and convergence plots (lower panel) for the 19 PPCs calculated using the DFE procedure.

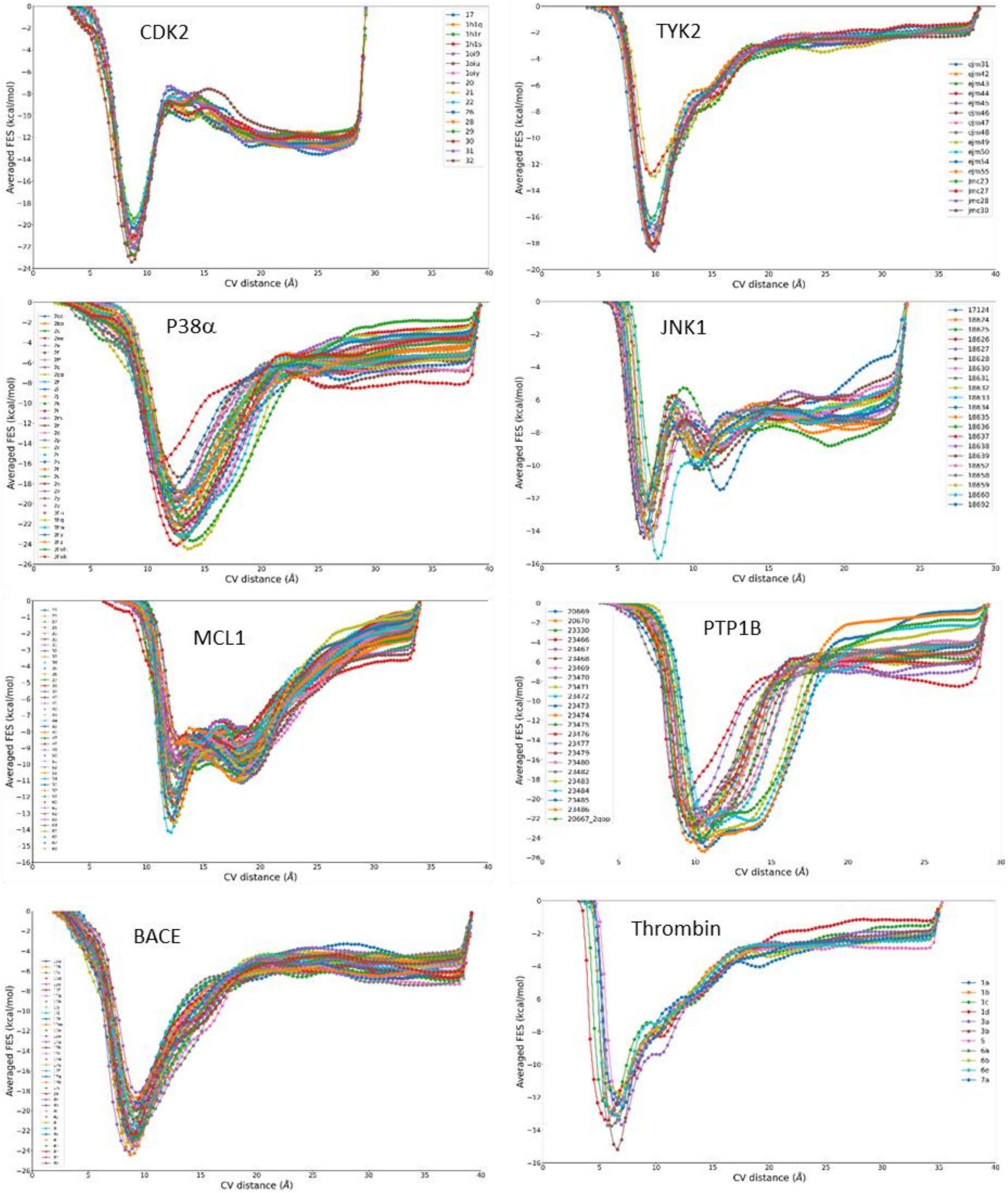

Supplementary Figure S2. Averaged FES of PLCs for each of the eight targets. Each curve corresponds to one PLC and the curves of different PLCs of a same target are grouped into one panel.

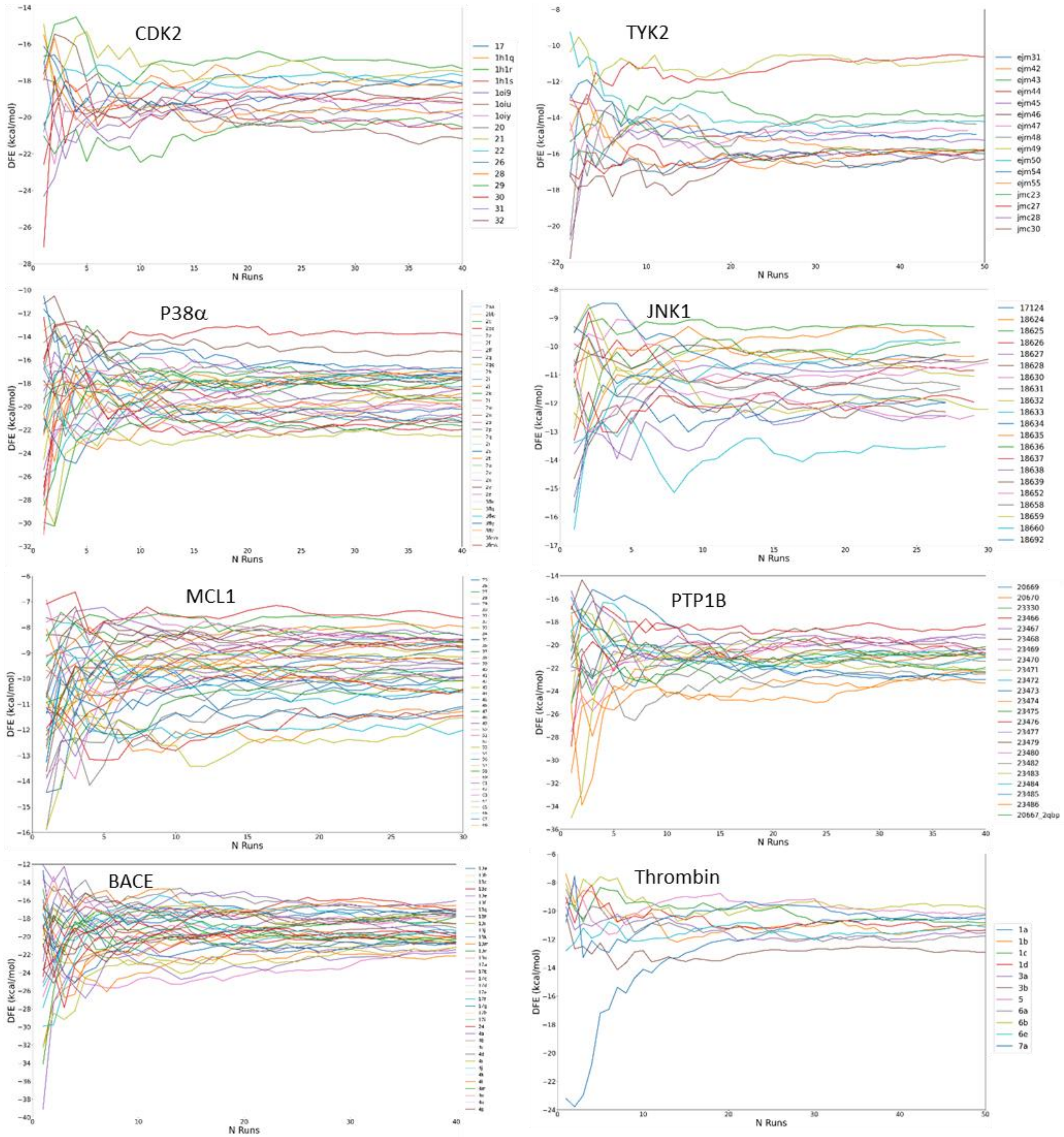

Supplementary Figure S3. Convergence plots of PLCs for each of the eight targets. Each curve corresponds to one PLC and the curves of different PLCs of a same target are grouped into one panel.

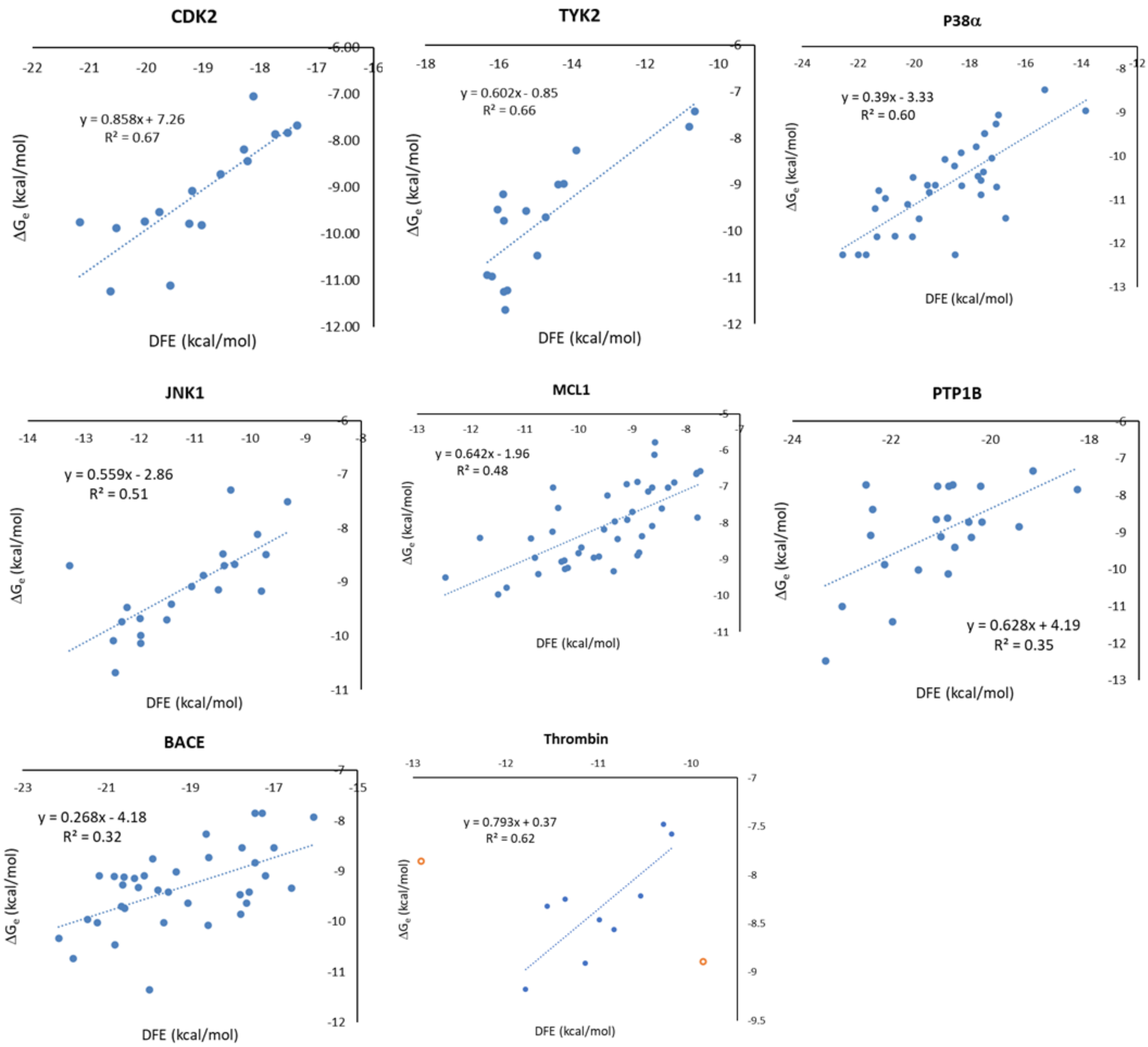

Supplementary Figure S4. Correlation plots between calculated DFE and experimental binding free energies ΔG_e. Each panel corresponds to a protein target with respect to a set of ligands.

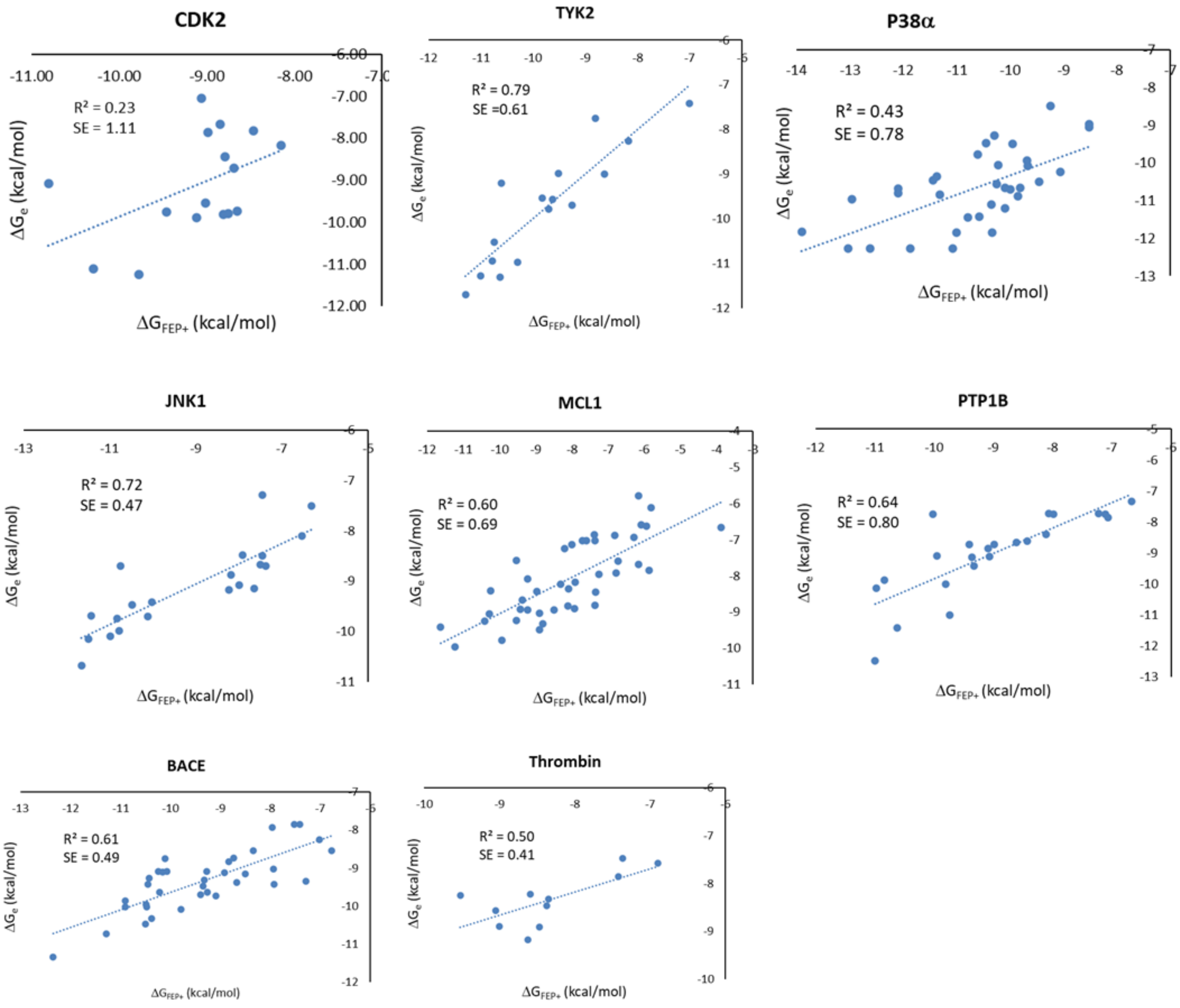

Supplementary Figure S5. Correlation plots between binding free energies ΔG_{FEP+} calculated using FEP+ program and experimental binding free energies ΔG_e. Each panel corresponds to a protein target with respect to a set of ligands. ΔG_{FEP+} were calculated based on $\Delta\Delta G_{FEP+}$. Both ΔG_{FEP+} and ΔG_e were taken from Wang, L. et al. *J. Am. Chem. Soc*. **137**, 2695−2703 (2015).
